# 1782. Spatiotemporal Trends in Group A Streptococcal Pharyngitis in the United States

**DOI:** 10.1093/ofid/ofad500.1611

**Published:** 2023-11-27

**Authors:** Madeleine Kline, Stephen M Kissler, Yonatan H Grad

**Affiliations:** Harvard T.H. Chan School of Public Health, Boston, Massachusetts; University of Colorado Boulder, Boston, Massachusetts; Harvard Chan School of Public Health, Boston, Massachusetts

## Abstract

**Background:**

Pharyngitis caused by group A *Streptococcus* (GAS) drives many outpatient pharyngitis visits and antibiotic prescriptions in the US and can have serious post-infection immunological sequelae. Like many other upper respiratory infections, GAS pharyngitis burden is usually highest in the winter. However, compared to other common illnesses such as RSV or influenza, relatively little is known about the spatiotemporal distribution of GAS pharyngitis in the US. We analyzed outpatient claims data to better characterize regional differences in the burden and seasonal timing of GAS pharyngitis across US census regions.

**Methods:**

US outpatient insurance claims from private insurers for GAS pharyngitis were extracted from the IBM MarketScan database from 2010-2018. Data were weighted to be representative of the overall population, and visits per month were averaged across the US census regions. Seasonal trends were assessed by fitting sinusoids to monthly state-, and region- level data using nonlinear-least squares regression.

**Results:**

The South had consistently more GAS pharyngitis visits per 1000 people compared to the other regions, and the West had fewer visits per 1000 people (South January Average: 3.78, 95% CI 3.36-4.21, West January Average: 1.76, 95% CI 1.62-1.90) (Fig. 1). The wintertime peak in visits began its rise earlier in the South than other regions, and the regional differences in visits were most pronounced from July through December. Annual GAS pharyngitis visits peaked earliest in the South, particularly the West and East South Central subregions, in February (West South Central peak month: 1.49, 95% CI 1.20-1.77; East South Central peak month: 1.50, 95% CI 1.22-1.79), then radiated outwards peaking last in March on the coasts (Pacific West peak month: 2.35, 95% CI 2.10-2.61; New England peak month 2.62, 95% CI 2.44-2.81) (Fig. 2).

Figure 1

**Figure:**
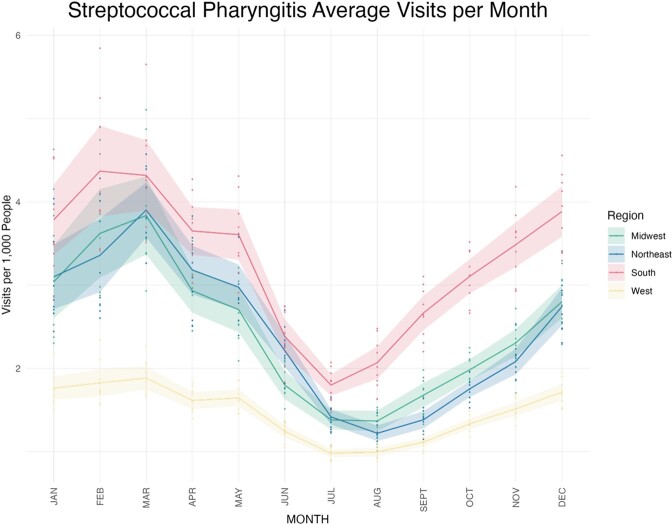

Figure 2

**Figure:**
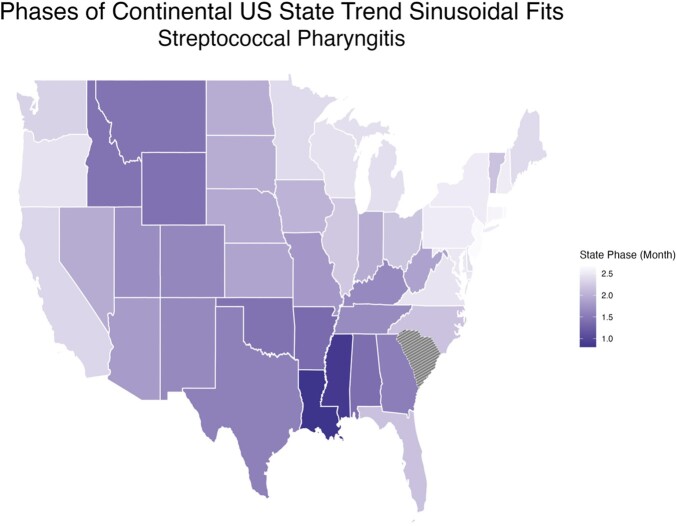

**Conclusion:**

GAS pharyngitis visits showed distinct regional trends in burden and seasonality. These regional differences could indicate where and when to expect the most severe disease, complications, and sequelae. Further investigation is needed to uncover the mechanism of these differences –including socio-behavioral, environmental, or circulating strain– and to target interventions to reduce burden of disease.

**Disclosures:**

**Stephen M. Kissler, PhD**, ModernaTx: Advisor/Consultant **Yonatan H. Grad, MD, PhD**, Day Zero Diagnostics: Board Member|GSK: Advisor/Consultant

